# Access to contraceptive services during the COVID-19 pandemic: clients’ perspective at primary health care level from India, Nigeria and Tanzania

**DOI:** 10.1186/s12978-025-02123-w

**Published:** 2025-09-07

**Authors:** Rita Kabra, Komal Preet Allagh, Tanimola Makanjuola Akande, Ester Elisaria, Beena Joshi, Adesola Olumide, Mary Ramesh, Donat Shamba, Deepti Tandon, Ranjan Prusty, Bhavya MK, Shabana Khan, James Kiarie

**Affiliations:** 1 Department of Sexual and Reproductive Health including UNDP/UNFPA/UNICEF/WHO/World Bank Special Programme of Research, Development and Research Training in Human Reproduction, World Health Organization, Avenue Appia 20, 1211 Geneva, Switzerland; 2 Department of Sexual and Reproductive Health and Research, World Health Organization, Geneva, Switzerland; 3 University of Ilorin, Ilorin, Nigeria; 4 Department of Health System, Impact Evaluation and Policy, Ifakara Health Institute, P.O. Box 78373, Dar es Salaam, Tanzania; 5 Department of Operational and Implementation Research, ICMR National Institute for Research in Reproductive and Child Health, Mumbai, India; 6 Institute of Child Health, University of Ibadan, Ibadan, Nigeria; 7 Department of Impact Evaluation, Health System and Policy Analysis, Ifakara Health Institute, Plot 463, Kiko Avenue Mikocheni, P.O. Box 78 373, Dar es Salaam, Tanzania; 8 Department of Clinical Research, ICMR, National Institute for Research in Reproductive and Child Health, Mumbai, India; 9 Department of Biostatistics, ICMR National Institute for Research in Reproductive and Child Health, Mumbai, India

**Keywords:** Family planning, Contraceptives, Barriers, COVID-19, Qualitative study, Primary health facilities

## Abstract

**Background:**

The COVID-19 pandemic disrupted the provision of sexual and reproductive health services, including contraceptive and family planning (FP) services. The World Health Organization conducted a multi-country study in India, Nigeria and Tanzania to assess the impact of the pandemic on the health system's capacity to provide contraceptive and FP services. In this paper, we share the results of a qualitative study aimed at understanding clients’ perspectives at the primary healthcare level on accessing contraceptive services in COVID-19-affected areas in the three aforementioned countries.

**Methods:**

We conducted interviews with 644 clients seeking contraceptive services across 11, 6 and 33 primary health facilities in India, Nigeria and Tanzania. A total of 44 focus group discussions (FGDs) and 128 in-depth interviews were conducted with clients at the facility and 22 FGDs within the community. Data collection took place from May 2022 to August 2022.

Ethical approval was obtained from the WHO Ethics Review Committee and national regulatory bodies. All interviews were analysed using the general approach of content analysis.

**Results:**

Clients at primary health care facilities faced several challenges in accessing contraceptive services. These challenges were grouped into two main categories. The first was related to the unprepared health system (supply), such as a shortage of health workers, stock out of contraceptives or high cost of FP services. The second category was outside the remit of the health system and included insufficient knowledge amongst clients about the availability of FP services, socio-cultural issues like spousal and in-laws’ dominance on decision making, restriction in movement due to lockdown and fear of COVID-19 infection.

**Conclusions:**

This study highlights the obstacles clients faced in accessing contraceptives during the COVID-19 pandemic in Nigeria, India, and Tanzania. To address these barriers in future crises, ministries of health must establish functional emergency preparedness across all healthcare levels. These plans should prioritize both on the sufficient number/gender of skilled health providers and the availability of contraceptives till the last mile. Utilizing e-health can help keep communities well informed on where, how and when to avail FP services during such emergencies. Health educational programs should actively engage men to gain further support.

## Abstract (plain language summary)

The COVID-19 pandemic disrupted many healthcare services, including sexual and reproductive healthcare, such as contraceptive and family planning services. Our study conducted in India, Nigeria, and Tanzania aimed to understand the impact of the COVID-19 pandemic on contraceptive and family planning services. This paper presents findings on the experiences of male and female clients accessing contraceptive services at primary health facilities during the pandemic across these countries. We interviewed 424 women and 220 of their male partners seeking contraceptive services in primary health facilities in the three countries between May and August 2022.

Clients at health facilities in all three countries faced many challenges in getting contraceptives. They faced problems like not enough staff at the facility, unavailability of contraceptives, fear of getting COVID-19 infection, not knowing where to find family planning services, increased cost of contraceptive services, no transportation to go to the facility or pressure from family members about decisions.

Health ministries need to plan better to tackle these issues in future emergencies. They need to make sure that there are enough trained staff and that contraceptives are available everywhere. Technology like e-health can help spread information about family planning services during emergencies. Also, it's important to educate men to get their support for family planning.

## Background

The World Health Organization (WHO) declared the coronavirus disease (COVID-19) as a pandemic on 11 March 2020 [1]. Studies indicate that the COVID-19 pandemic had a major impact on the availability and provision of contraception, abortion services, post-abortion care, and other sexual health services [2–4]. The WHO Pulse survey (round 1) conducted between May and July 2020 showed that 68% of 102 countries experienced disruptions in FP and contraception services [5]. In response, WHO geared up to support countries to adapt national systems to continue access and coverage of essential services including sexual and reproductive health (SRH) and family planning (FP) services based on generic guidance [6–9]. However, despite some evidence of service restoration, significant disruptions to FP and contraceptive services persisted over a year into the pandemic. Round 2 of the pulse survey indicated that 44% of 104 countries still reported disruptions [10]. The unmet family planning needs during this time could lead to increased unintended pregnancies, with long-term consequences for both women and their families [11]. The Guttmacher Institute estimates that a 10% reduction in essential SRH services because of the pandemic could result in an estimated 15 million additional unintended pregnancies, 3.3 million unsafe abortions, and 29,000 additional maternal deaths [11–13].

The first case of COVID-19 was reported in India on 20 January 2020 [14], in Nigeria on 27 February 2020 [15] and in Tanzania on 16 March 2020 [16]. India and Nigeria implemented lockdown measures in the last week of March 2020 [17, 18], while Tanzania did not enforce the official lockdown.

Several factors impacted access to family planning and contraceptive services during the pandemic, including supply chain disruptions [19], reduced healthcare facility services [20], reassignment of healthcare providers to COVID-19 duties [21], lockdowns or fear of COVID-19 [22], movement restrictions and shortages of health workers [23].

We conducted a mixed methods study to evaluate the impact of the COVID-19 pandemic on the primary health system’s capacity to provide FP and contraceptive services and the perceptions of clients and healthcare providers about the access and use of FP and contraceptive services in India, Nigeria and Tanzania [24]. This study included in-depth interviews and focus group discussions to explore clients’ and healthcare providers’ perspectives on FP and contraceptive service availability. Additionally, a cross-sectional health facility assessment was conducted to evaluate the health facility infrastructure’s ability and readiness to provide FP and contraceptive services and trends in service availability during the COVID-19 pandemic. In this paper, we present findings of the barriers encountered by women of reproductive age and their male partners in accessing contraceptive services at primary health facilities in India, Nigeria and Tanzania. Unlike existing studies, which often have smaller sample sizes and do not specifically focus on primary healthcare facilities, our research delved into the primary healthcare level. Furthermore, we explored the barriers men face in accessing FP services, a dimension often overlooked in previous research studies. In addition to examining challenges within primary health facilities, we also investigate the barriers women in the community encounter when accessing FP services. This comprehensive approach allowed for data triangulation, providing a more nuanced understanding of the obstacles to contraceptive access and utilization across different settings.

## Methods

This qualitative study was conducted in India (IN), Nigeria (NIG) and Tanzania (TAN) between May 2022 and August 2022. These countries were selected based on the willingness of the Ministries of Health to participate in the study and the research institutions’ capability and capacity in the respective countries to conduct the study. The Indian Council of Medical Research- National Institute of Research in Reproductive and Child Health (ICMR NIRRCH) in India, the University of Ilorin Teaching Hospital in Nigeria and the Ifakara Health Institute in Tanzania led the study.

### Study setting

The study locations within each country were selected based on specific criteria, including geographic accessibility, availability and organization of family planning services, areas with high COVID-19 incidence rates, and where the pandemic was likely to have significantly impacted service delivery. Primary healthcare facilities offering contraceptive services and staffed with qualified personnel to provide contraceptive services were selected for the study. Both rural and urban representation of the primary health facilities was ensured. It was expected that the variation in the distribution of primary health facilities within geographical areas would provide representation to communities of all socioeconomic backgrounds. In total, 11, 6 and 33 health facilities from India, Nigeria and Tanzania, respectively, were included in the study.

### Study design

A qualitative approach was applied using in-depth interviews (IDIs) and focus group discussions (FGDs). IDIs and FGDs were conducted with women of reproductive age (18-49 years) who had sought or received contraceptive services from the selected primary health facilities and were comfortable discussing their experiences of seeking contraceptive services in group settings (for FGDs) and their male partners. Male partners either accompanied the females to the clinic or the females assisted with recruiting them. The participants will be purposively selected to obtain a sample of women/girls and their partners from all socioeconomic backgrounds in the area. We conducted FGDs with women in the community who had not received contraceptive services from the selected health facilities but may have sought them from other private or public health facilities.

Health providers not involved in the study (gatekeepers) assisted in identifying potential participants based on selection criteria and confirmed their willingness to participate. Screening was done as an exit interview after the women had been attended to or while the women were waiting to be attended and were taken to a private room selected by the gatekeeper and the study team. Clients were screened privately in a room approved by the study team. Clients agreeing to participate were introduced to the research team and provided comprehensive information about the study. Interviews continued until saturation was reached. Clients were recruited for IDIs and FGDs, until thematic saturation.

IDIs and FGDs were guided by prepared topic guides and were conducted in a convenient and safe place for participants. The original questionnaire was prepared in English and translated into 5 languages: Yoruba, Hausa (in Nigeria), Kiswahili (in Tanzania), Hindi and Marathi (in India). The IDIs lasted between 40 and 60 minutes, and each FGD lasted between 60 and 90 minutes. All interviews were recorded with participants’ consent obtained in writing.

### Data analysis

After completing all interviews, the recorded interviews were transcribed verbatim in their original language, then translated to English and back-translated to ensure accuracy. Data was analyzed using a general content analysis approach, following steps outlined by Elo and Kyngäs [25]. Initially, the transcripts were reviewed multiple times to understand their content and develop a categorization matrix (preparation phase). In the organization phase, primary codes were identified in the manuscripts. These codes were then categorized based on similarities and differences and subsequently organized into sub-themes. Finally, sub-themes were grouped under the main themes. As a result, themes, the reporting phase presents the identified themes and sub-themes that highlight the barriers clients faced in accessing contraceptive services across the three countries. During data analysis, two researchers were used to check the inter-coder reliability of the categorization. Two researchers independently analyzed the same dataset using Nvivo software (version 12; QSR International).

### Ethical considerations

The study obtained ethical approval from the WHO Ethics Review Committee (Protocol IDs CERC.0103K, CERC.0103J and CERC.0103I). Additionally, approval was granted by the Institutional Ethical Committee (IEC) of ICMR NIRRCH, Mumbai (D/ICEC/Sci-166/175/2021), state and district health officials of selected sites in India, the National Health Research Ethics Committee of Nigeria (NHREC/01/01/2007-07/09/2021), the University of Ilorin Ethical Research Committee (Kwara state) and State Ministries of Health Ethical Committee (Kwara, Kano and Oyo States) in Nigeria, the Ifakara Health Institutional Review Board (IHI/IRB/NO. 44-2021), the National Institute of Medical Research (NIMR/HQ/R.8a/Vol.IX/3916) and the President’s Office, Regional and Local Government (PORALG) in Tanzania. Written informed consent was obtained from all participants.

## Results

### Participant characteristics 

Sixty-six focus group discussions and 128 in-depth interviews were conducted across the three countries (Table 1). These included 644 participants, with 264 from India, 242 from Nigeria, and 138 from Tanzania. Among the participants, 65.8% (*n *= 424) were women. The majority of participants were married (97.4%, *n* = 627). One-third of the participants (32.5%, *n* = 209) had attained education levels up to secondary education. Further details regarding the socio-demographic characteristics of the participants can be found in Table 2.

**Table 1:** Overview of FGDs and IDIs conducted in the countries

	**FGD in facility**		**FGD in community**	**IDIs**	
	**Women**	**Men**	**Women**	**Women**	**Men**
**India**	9	9	9	26	13
**Nigeria**	7	7	7	34	34
**Tanzania**	6	6	6	15	6
**Total**	**44**		**22**	**128**

**Table 2:** Socio-demographic characteristics of participants

**Participants characteristics**	**India (*n *= 264)**	**Nigeria (*n* = 242)**	**Tanzania (*n* = 138)**
**Gender, n (%)**			
Female	185 (70.1%)	147 (60.7%)	92 (66.7%)
Male	79 (29.9%)	95 (39.3%)	46 (33.3%)
**Age, mean (SD)**	31 (±5.201)	34.4 (±10.9)	32 (± 7.5)
**Educational level, n (%)**			
None	7 (2.7%)	17 (7%)	13 (9.4%)
Primary	18 (6.8%)	47 (19.4%)	79 (57.2 %)
Secondary	47 (17.8%)	121 (50%)	41 (29.7%)
High school	130 (49.2%)	27 (11.2%)	2 (1.4)
University	31 (11.7%)	27 (11.2%)	3 (2.2%)
Graduate school	31 (11.7%)	3 (1.2%)	0
**Marital status, n (%)**			
Single	0	0	0
Married	264 (100%)	241 (99.6%)	122(88.4%)
Divorced	0	1 (0.4%)	8 (5.8 %)
Cohabitation	0	0	8 (5.8 %)

### Main extracted themes

**Fig. 1:**
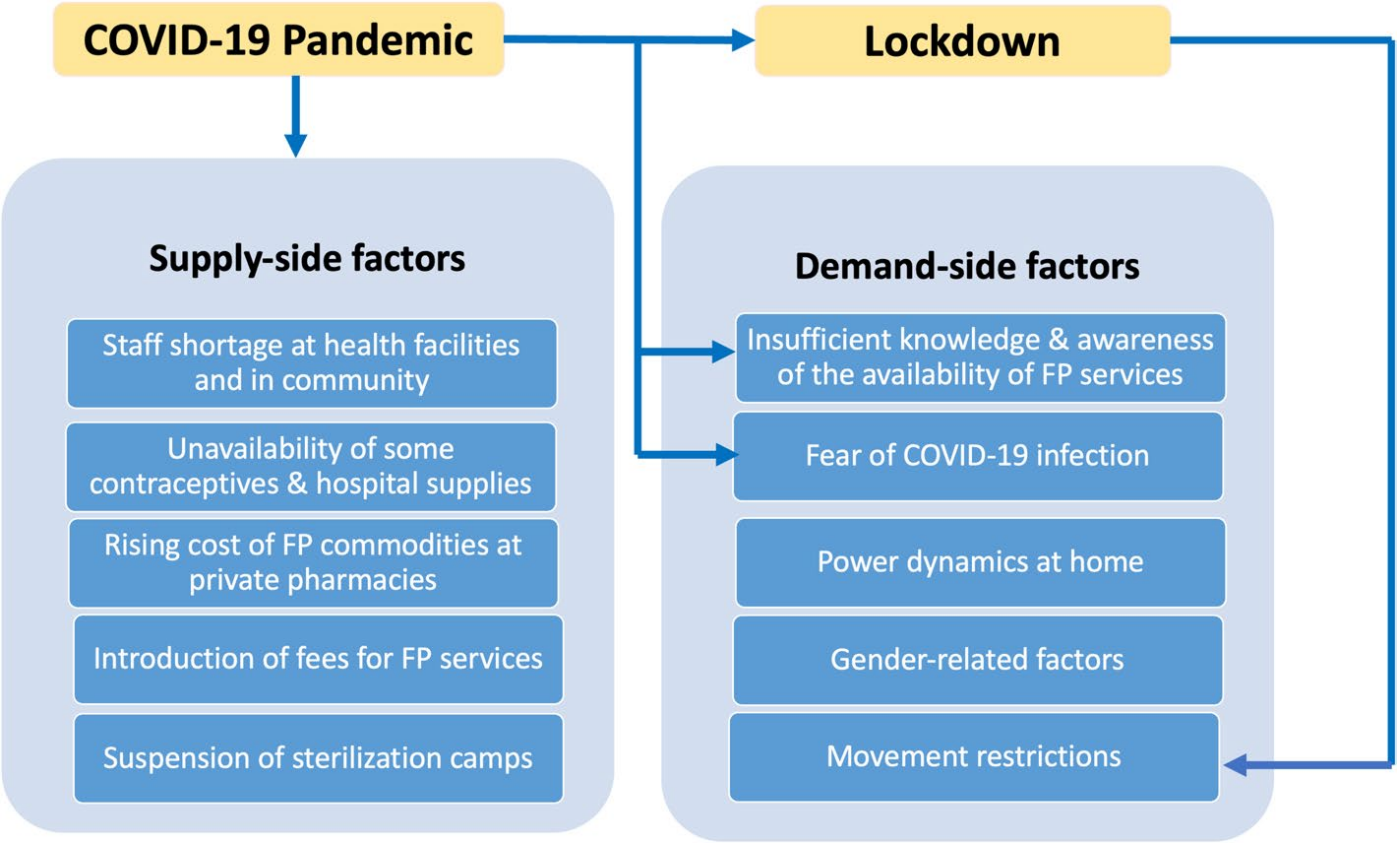
Overview of themes and sub-themes summarizing the barriers faced by clients in accessing FP and contraceptive services during the COVID-19 pandemic

The barriers identified by clients in accessing contraceptive services were categorized into ten subthemes distributed among two main themes (Fig. 1): (i) Supply-side factors (staff shortage at health facilities and in community, unavailability of some contraceptives & hospital supplies, rising cost of FP commodities at private pharmacies, introduction of fees for FP services, suspension of sterilization camps) and (ii) Demand-side factors (insufficient knowledge and awareness of the availability of FP services, fear of covid-19 infection, power dynamics at home, gender-related factors, movement restrictions due to lockdown). These themes highlight the multifaceted challenges individuals, communities and health facilities faced in accessing and providing contraceptive services across the study locations.

## Supply-side Factors

### Staff shortage at health facilities and in the community

Nigeria and Tanzania highlighted the shortage of healthcare providers at facilities, which limited the number of clients who could be served effectively. A participant from Nigeria expressed, “*Staff were available, but they were not as sufficient as before, so this delayed them from attending to a larger number of people unlike before the pandemic” (NIG- OY_FGD_FWC_Urban).* Another participant shared, *“There are only a few people that go to the hospital. You may go to the facility, but you will not be able to see a doctor. Women go for ANC. Some were attended to while some are not…” (NIG- KN_FGD_ female community member _03_URBAN).* A male participant from Tanzania mentioned, *“There was a challenge because they limited the number of clients coming to the health facility. For example, they will tell you that they will only attend to ten patients, so the other patients who came for that service will be told to come back on the next day” (TAN-P1, male client, FGD 7, Moshi MC).* Furthermore, respondents noted that the attitude of health workers posed a major challenge in accessing services at the facilities. One participant from Nigeria expressed, *“The barrier in any government health center or hospital for those using them is the attitude of our health workers who attend to us if one is not patient he will not wait to be attended to. That is the first problem.” (NIG- KW_FGD_MP_U).* A participant from Tanzania shared, *“When we went to the facility, we were told to come back the next day, when you go back the next day they tell you to come back another day” (TAN-P5, female client, FGD 3, Nyamagana MC).*

Shortage of community health workers was a pressing issue voiced by participants from Nigeria, who expressed concerns about the absence of these workers and the subsequent lack of contraceptive provision. One participant shared*, “We could not obtain family planning services during the pandemic because there was no one to come to our community as they usually do to advertise the family planning and give it to us.” (NIG-OY_FGD_FP User_Urban)*

### Unavailability of some contraceptives & hospital supplies

Shortages of condoms and oral contraceptive pills were reported during the pandemic. Some participants mentioned that contraceptive services were accessible in rural public facilities during COVID-19, while urban facilities experienced shortages. However, participants also noted that these shortages were temporary. Respondents from Nigeria and India raised the issue of the unavailability of preferred contraceptive methods at health facilities. A participant from Nigeria expressed, *“Sometimes when we come here, we are unable to get all the services because they may say a particular family planning method is unavailable” (NIG-OY_FGD_FPusers_Urban)*. An interviewee from India shared, *“Sometimes stock is not available with CHWs, but they restocked soon and informed the community. Two to three times, it happened, and they restocked it in two to three days. It happened especially during the first wave”. (IN-IDI women rural).* Clients seeking long-term contraceptive methods in Tanzania were required to purchase items for the procedure*.* A female participant shared, *“There was a challenge with equipment; we were told to buy gloves” (TAN- P5, female client, FGD 3, Nyamagana MC).* Another participant mentioned, *“When you come here at the facility for implanon, they ask you to go and buy gloves, so I thought that gloves were not available. If you don’t have gloves, you won’t receive services. Also, sometimes when you go, you are told anaesthetic drugs are not available” (TAN-P4, female client, FGD 5, Nyamagana MC).*

### Rising cost of FP commodities at private pharmacies

A few respondents from India reported that the cost of contraceptives was high at private medical stores. *“The cost was approximately Rs. 200 per packet of oral contraceptive pills from private medical shops, which was high” (IN_IDI women, Urban).* Another participant stated *“The cost of condom was also increased from Rs. 25 to 50 at time of lockdown at private medical shops” (IN_IDI women, Rural).* Thus, this added to the barriers clients faced in accessing contraceptive services.

### Introduction of fees for FP services

Clients from Tanzania and Nigeria expressed concern about being charged for contraceptive services during the pandemic, which were previously provided free of charge. A participant from Nigeria shared, *“They were doing it free of charge, we don't know why they stopped” (NIG-OY_IDI_FPUser_05_Rural).* Another participant stated, *“I don’t have access to it because we were told it’s not available except, we buy, and I don’t have the money” (NIG- OY_FGD_WRA_Urban).* Similarly, a client from Tanzania recounted a friend’s experience, saying, *“My friend told me about the challenge she faced when she came to seek contraceptive services. Since she was experiencing discomfort with the implant, she wanted to shift to another method, but health care providers asked her to pay so as to receive the services, while we know that contraceptive services are supposed to be provided for free” (TAN-P5, male client, FGD 2, Nyamagana MC).*

### Suspension of sterilization camps

Tubal ligation camps, were suspended at some facilities in India, thereby preventing access to FP services. *“Actually, our son is 5 years old, so we planned for going for FP surgery and even insertion of CU-T was in our plan, but due to covid it got postponed” (IN_FGD men, rural).*

## Demand-side factors

### Insufficient knowledge and awareness of the availability of FP services

The lack of sufficient information about the availability of contraceptive services at health facilities was a significant concern raised by respondents from India and Nigeria. In India, participants, particularly men in urban areas, expressed uncertainty about whether FP services were still provided during the COVID-19 imposed lockdown/restrictions. This lack of awareness contributed to hesitancy in seeking contraceptive services. “*We were not aware if the contraceptive services are provided at the facility, as everything was closed, we thought even these services might not be available*” (IN, FGD men, urban).

### Fear of COVID-19 infection

Fear of contracting COVID-19 significantly deterred participants from seeking contraceptive services across all three countries. In India, urban men expressed reluctance to seek sterilization services, due to apprehensions about COVID-19. *“Yes, we wanted to get the operation done, but due to fear of COVID, we did not plan for it.*” *(IN-FGD men, urban).* In Nigeria, women in urban areas reported refusing to visit health facilities because of fear of COVID-19. *“During the pandemic many women refused to go to the health facility because what they heard was, once you have catarrh then it means you have COVID” (NIG-KN_FGD_ female community member _03_URBAN).* Furthermore, women in Nigeria mentioned that they avoided contraceptive services due to concerns about going out during the pandemic. *“The one I use is important but couldn’t get it then because I was scared to go out” (NIG-OY_IDI_FPusers_02_Urban*). Notably, some participants in India mentioned that the mandatory COVID testing required before receiving treatment discouraged them from visiting the health facilities for contraceptive services. “*Also, first the COVID test was done before giving the treatment that was another reason that people did not visit the facility.*” *(IN-FGD men, rural).* In India, there were even concerns about purchasing contraceptives online due to rumours about COVID-19 transmission through purchased items. “*During Covid pandemic, many rumours were spread that if any items purchased from outside must be avoided or used after proper sterilization as they could also transmit infection as handled by many people who may have had Covid infection.*”

### Power dynamics at home

Women from India and Nigeria emphasized the need for permission from their partners and/or in-laws’ to access contraceptive services. One participant shared, “*My husband and mother-in-law did not permit me to take an injection and Copper-T insertion, but I decided to take an injection, and I started taking the injection in September 2020*” (IN-FGD women, rural). Another woman expressed, “*My husband and mother-in-law didn’t allow using contraception*” (IN-FGD women not availing, urban). In Nigeria, it was noted, *“it is the father that makes a final decision to go to the facility being the head of the family. Next in line is the mother.” (NIG-KN_FGD_FWC_03_URBAN).* Partners sometimes prohibited the use of certain contraceptive methods, such as the Copper-T IUD. A participant explained, “*I did not get Copper-T inserted, as it is of metal, I had heard that women had got copper t inserted, but during sexual contact, the copper got stuck on the penis, which had to be removed on an emergency basis. This caused a lot of humiliation, and sadly, we heard the woman committed suicide after some days. So, my husband is against Copper-T. He doesn’t allow me that option” (IN- FGD women, not availing, urban).*

### Gender-related factors

Male participants from India expressed discomfort in approaching female community health workers (ASHA workers) for condoms. One participant highlighted, *“The main thing is we can ask for sanitizer from a woman, but we cannot ask for a condom as easily as we could have asked a man, so government should think of some alternative so that men can easily have access to condoms” (IN-FGD, men, rural).* Additionally, some men emphasized the need for a male helper at the medical shops within health facilities *“There should be more awareness; there should be a male helper at the facility as men hesitate to talk openly to the female staff at the facility” (IN-FGD men, urban).*

### Movement restrictions due to lockdown

Lockdowns during the COVID-19 pandemic led to movement restrictions, which posed significant challenges for clients across from all three countries in accessing contraceptive services. Participants reported the non-availability of transportation as a major barrier. One male participant from a rural area told us that he abstained from sexual activity due to the inability to access condoms because of transportation issues. He shared *“We did not face problem in getting it, but as transport was not available and police were stopping us and we couldn’t tell them that we are going out to buy a condom*” *(IN-FGD men, rural).* Another female participant from Nigeria mentioned, *“There is no major change, but the number of clients has reduced. Not everyone makes it to the facility due to lack of commercial vehicles.” (NIG-KN_FGD_female community member_03_URBAN).* Similarly, a participant from Tanzania highlighted the challenges of overcrowded buses and the need to walk long distances to reach the hospital due to transportation issues*: “There are challenges because you might enter in the bus, but you find that people are overcrowded so you have to walk even a long distance to reach the hospital. I used to live far and the buses there leave very early, so I have to look for money to hire a motorcycle” (TAN-P1, female client, FGD 10, Moshi MC).*

## Discussion

Our study identified that men and women faced several barriers to accessing contraceptive services during the COVID-19 pandemic in India, Nigeria, and Tanzania. The predominant barrier across all three countries was movement restrictions and no transport available to take people to the facility. Additionally, clients faced challenges such as shortage of contraceptive supply, shortage of staff, insufficient knowledge on the availability of contraceptives, fear of COVID-19, and influence of family on decision making, particularly in India and Nigeria. Another significant barrier observed was the introduction of fees for contraceptive services in Nigeria and Tanzania, which were previously free. This reduction in access and use of FP and contraceptive services and a surge in health risks associated with unplanned and unintended pregnancies contribute to higher infant and maternal mortality rates.

A comparative study examining the healthcare providers'and clients'experiences in accessing FP and abortion services in Bangladesh, Iran and the Netherlands identified reduced referrals, disrupted access, insufficient knowledge, staff concerns, rising prices and contraceptive unavailability as key challenges [26]. Movement restrictions, the need for spousal permission, contraceptive stock outs and shortages of health workers were also reported as challenges in a qualitative study among women in Nepal [27]. In Bangladesh, fear and stigma related to COVID-19 emerged as the main barrier to FP uptake [2], while in India, movement restrictions, fear of COVID-19 and suspension of tubal ligation services have posed challenges [28]. A cross-sectional study in Nigeria revealed fear of visiting health facilities, closed medical shops, restricted movement and lack of access to healthcare providers as the major obstacles to contraceptive access during the lockdown [29]. Barriers to contraception access in developing countries include contraceptive shortages due to supply chain disruption, reduced priority to FP counselling, clinic closures, fear of COVID-19, non-availability of health staff and limitation of transportation [30]. A study conducted across 14 countries under the WHO FP Accelerator project in 2020 identified reduced client demand, closure of health facilities, shortages of FP commodities and supplies, anxieties among health workers and disruptions to FP outreach services as primary barriers limiting access to FP services during the pandemic [31]. A scoping review summarized key barriers to accessing FP services as fear of infection, lack of transport, travel-related restrictions, cost, increased waiting time, limited home visits by health workers, and lack of supplies [2]. Moreover, the gender of the community health workers has emerged as a barrier to accessing contraceptive services during the pandemic in our study, a factor not previously reported. This discrepancy may be attributed to earlier studies primarily focused on female participants, resulting in male perspectives being overlooked. In our study, conversations with male clients specifically highlighted the gender of community health workers as a barrier to accessing FP services. A systematic review (before the pandemic) showed that the sex of the providers affected the acceptability and uptake of services in the community [32]. A qualitative research study from Kenya has shown that the lack of male CHWs is perceived by the community as a barrier to effective family planning [32].

The issue of male partner control as a barrier to contraceptive use persists regardless of the COVID-19 pandemic. Lockdowns and social consequences of COVID-19, such as increased financial strain, potentially exacerbate this challenge [33]. Men’s resistance to family planning, reinforced by numerous gender norms linking men’s status to large family size, remains one of the most salient barriers to women’s contraceptive use. Our study also revealed similar barriers in Nigeria and India. As a result, women who rely on contraceptives without their partners’ knowledge would face difficulty in accessing these services, especially when their partners are present at home during lockdowns, thereby increasing the risk of unplanned pregnancies.

Limitations and bias mitigation: While our study provides valuable insights into the pandemic’s impact on contraceptive service access, its geographic coverage and the number of healthcare facilities examined are limited, potentially affecting the generalizability of findings.

The potential risk of bias associated with using gatekeepers to recruit clients for IDIs and FGDs was minimized by ensuring that the gatekeeper’s role was strictly limited to identifying and introducing potential participants to the research team. To further reduce bias, the study team emphasized to gatekeepers the importance of identifying all eligible participants, ensuring inclusivity and representation. Specific screening questions were provided to standardize participant selection and prevent unintentional selection bias.

Nonetheless, the study highlights crucial challenges posed by the pandemic.

Based on our study findings, we recommend policymakers and ministries of health to implement the measures outlined in Table 3.

**Table 3:** Key policy recommendations for strengthening FP services during a health crisis

**Recommendation**	**Description**
1. Inclusion in emergency preparedness plans	Ensure that all emergency, epidemic or pandemic preparedness plans integrate access to a comprehensive range of contraceptives. The participation of SRH representatives in administrative structures designed to mitigate the effects of the pandemic is important.
2. Dispelling misinformation and promoting confidence in healthcare facilities	Address misconceptions and build trust in healthcare facilities to encourage contraceptive use. Providing accurate information and promoting positive experiences with healthcare services can help alleviate fears and increase the utilization of FP services.
3. Enhanced Community knowledge and awareness	Regular advocacy and awareness campaigns should inform communities about service locations, operating hours, and available contraceptive methods, empowering individuals to make informed reproductive health choices.
4. Strengthen alternative service delivery channels	Use telemedicine, digital health, mobile clinics, and community outreach to mitigate service disruptions and ensure continuity of care, especially in remote areas. These options also offer privacy and convenience.
5. Strengthen the supply chain system	Ensuring adequate contraceptive stock at primary health facilities for individuals to access their preferred methods. Standardized national FP and contraceptive services guidelines should be developed to ensure FP service continuity during a health crisis.

## Conclusions

This study has shed light on the substantial barriers hindering clients’ access to contraceptive services amidst the COVID-19 pandemic in Nigeria, India, and Tanzania. To overcome and avoid these barriers in future crisis, ministries of health need to ensure that the emergency/pandemic preparedness plan includes access to contraceptives and FP focusing both on a sufficient number/gender of skilled health providers and a functional supply chain system to ensure availability of contraceptives till the last mile. Emerging technologies like e-health and leveraging digital tools should be adapted to keep communities at all levels of care well informed on where, how and when to avail FP services during such emergencies. Implementing these strategies may enable girls, women, men, and others, to access the services they need to fulfil their reproductive health needs which can help governments to achieve their Sustainable developmental goals and Universal Health care for all.

## Data Availability

All data relevant to the study are included in the article or are available on request

## Abbreviations

COVID-19: Coronavirus disease 2019
FP: Family planning
FGD: Focus group discussion
ICMR NIRRCH: Indian Council of Medical Research National Institute of Research in Reproductive and Child Health
LMICs: Low and middle-income countries
IDI: In-depth interviews
IEC: Institutional Ethical Committee
mCPR: modern contraceptive prevalence rate
SRH: Sexual and reproductive health
WHO: World Health Organization
UNFPA: United Nations Population Fund
